# Neonatal Outcomes of Term Infants Born with Meconium-Stained Amniotic Fluid

**DOI:** 10.3390/children10050780

**Published:** 2023-04-26

**Authors:** Carlo Dani, Martina Ciarcià, Vittoria Barone, Mariarosaria Di Tommaso, Federico Mecacci, Lucia Pasquini, Simone Pratesi

**Affiliations:** 1 Division of Neonatology, Careggi University Hospital, Largo Brambilla, 3, 50141 Florence, Italy; 2 Department of Neurosciences, Psychology, Drug Research and Child Health, University of Florence, 50121 Florence, Italy; 3 School of Midwifery, University of Florence, 50121 Florence, Italy; 4 Department of Health Sciences, Section of Pediatrics, Obstetrics and Gynecology and Nursing, 50139 Florence, Italy; 5 Department of Clinical and Experimental Biomedical Sciences, Careggi University Hospital, University of Florence, 50121 Florence, Italy; 6 Fetal Medicine Unit, Department for Women and Children Health, Careggi University Hospital, 50134 Florence, Italy

**Keywords:** meconium-stained amniotic fluid, staging, outcome, infant

## Abstract

**Background** Meconium-stained amniotic fluid (MSAF) is considered an alarming sign of possible fetal compromise and it has recently been reported that neonatal outcome correlates with the degree of meconium thickness. **Methods** We retrospectively studied 400 term infants allocated in clear amniotic fluid and grade 1, 2, and 3 MSAF groups on the basis of color and thickness of AF. Multivariable logistic regression analysis was performed to evaluate the potential independent effect of delivery with MSAF of different severity on the risk of a composite adverse neonatal outcome. **Results** We found that delivery with grade 2 (OR 16.82, 95% Cl 2.12–33.52; *p* = 0.008) and 3 (OR 33.79, 95% Cl 4.24–69.33; *p* < 0.001) MSAF is independently correlated with the risk of adverse neonatal outcome, such as the occurrence of at least one of the following: need of resuscitation in the delivery room, blood cord pH < 7.100, occurrence of meconium aspiration syndrome (MAS), persistent pulmonary hypertension (PPH), transient tachypnea of the newborn (TTN), acute respiratory distress syndrome (ARDS), hypoxic-ischemic encephalopathy (HIE), and sepsis. **Conclusions** There is a positive correlation between the severity of amniotic fluid meconium staining and thickness and the outcomes of term infants. Therefore, the evaluation and grading of MSAF during labor is useful in order to plan for the presence of a neonatologist at delivery for immediate and proper neonatal care.

## 1. Introduction

Meconium-stained amniotic fluid (MSAF) is considered an alarming sign of possible fetal compromise associated with a poor perinatal outcome. The condition occurs in 7–20% of full-term births of women considered to be at normal risk [1], but only 2–4% of these deliveries are associated with the development of meconium aspiration syndrome (MAS), more than 90% of which are associated with thick MSAF [2]. MAS contributes to neonatal death in up to 0.05% (i.e., 1 in 2000 of all pregnancies) [3], but MSAF has also been associated with other neonatal morbidities, such as acute respiratory distress syndrome (ARDS) and hypoxic-ischemic encephalopathy (HIE) [4].

The volume of meconium in the fetal gut increases during the third trimester, and the amniotic fluid remains clear because the internal and external anal sphincters are closed during fetal life [5,6]. Therefore, MSAF is uncommon before the 38th week of gestational age, while its frequency progressively increases during gestation and is six times greater among women at 42 weeks or more compared with those at 37 weeks (18 vs. 3%) [7]. 

The development of MSAF may be the result of physiological maturation and motility of the fetal gastrointestinal tract but may also represent the fetal response to hypoxic stress [6]. In fact, fetal hypoxia can promote the release of arginine vasopressin from the fetal pituitary which stimulates the smooth muscles of the colon favoring the onset of hyperperistalsis and the relaxation of the anal sphincter [6]. However, also vagal (parasympathetic) stimulation from umbilical cord compression can induce an increase of peristalsis and relaxation of anal sphincter leading to intrauterine passage of meconium with [8] or without [6] fetal hypoxic stress. Finally, MSAF may rarely be due to intrauterine infection or biliary vomiting in utero secondary to fetal intestinal obstruction [6]. 

On the other hand, MSAF has been associated with several maternal predisposing factors, such as placental insufficiency, gestational diabetes, hypertensive disorders of pregnancy, oligohydramnios, and drug abuse (i.e., tobacco, cocaine), which can favor fetal hypoxia [9]. Therefore, anamnestic recognition of these conditions is of paramount importance to anticipate and identify which neonates could suffer morbidities associated with MSAF and, in that case, allow for proper neonatal resuscitation and postnatal care.

It has recently been reported that the outcomes of infants born through MSAF independently correlate with the degree of meconium thickness [10,11,12,13,14,15], but the literature on this issue is scarce and most studies are outdated, not very detailed, or with incomplete data analysis [10,11,12,13,14,15]. Moreover, it is possible that MSAF-related neonatal morbidities differ between different birth centers and neonatal intensive care units (NICUs), depending on the management of pregnancies, labor, and neonates.

Thus, on the basis of previous considerations, we hypothesized that there was a positive correlation between the different degree of MSAF and the risk of developing a composite adverse outcome in term infants. To test this hypothesis, we undertook this study with aim of correlating the delivery with MSAF of different thicknesses with the risk of developing a composite adverse neonatal outcome. 

## 2. Materials and Methods

### 2.1. Study Population

This single-center, retrospective study was carried out at Careggi University Hospital of Florence (Italy) following approval by the ethics committee. Infants with gestational age ≥37 weeks were studied if they were delivered after a trial of labor. Exclusion criteria were major fetal malformation, cases with bloody amniotic fluid, and elective cesarean deliveries. In our hospital, about 3000 newborns are born per year and the cesarean section rate in 2021 was 28%; there are 24 beds for neonatal special care (SP) and 10 beds for NICU. A neonatologist was present in all deliveries with MSAF and performed an immediate assistance of the newborn as needed. Further treatment and possible admission to neonatal units were decided according to clinical judgment.

### 2.2. Study Design

The study cohort was divided according to the grading of meconium staining into four groups: clear amniotic fluid (control group); grade 1 or light meconium group, when amniotic fluid is translucent and light yellow–green in color; grade 2 or intermediate meconium group, when amniotic fluid is opalescent with color between that of grades 1 and 3; grade 3 or heavy meconium group, in presence of a thick and opaque MSAF, deep green in color with visually identified particulate matter (the amniotic fluid “pea soup”) [6,10,13]. MSAF and its grading were always assessed by a midwife who reported each case to an obstetrician (including residents).

In cases of transitioning of MSAF thickness during labor, the highest level of meconium staining was reported. 

The first 100 infants consecutively born from January to November 2021 with clear amniotic fluid or with grade 1, 2, or 3 MSAF were assigned to the respective study groups. 

### 2.3. Data Collection

For each studied infant, we recorded gestational age, birth weight, intrauterine growth restriction (IUGR), sex, Apgar score at 1 and 5 min, need of resuscitation in the delivery room, blood cord pH, base excess (BE) and lactate level, admission in neonatal special and intensive care units, occurrence of MAS, persistent pulmonary hypertension (PPH), transient tachypnea of the newborn (TTN), acute respiratory distress syndrome (ARDS), air leak, hypoxic ischemic encephalopathy (HIE), sepsis, jaundice requiring phototherapy, and duration of hospital stay. MAS was diagnosed in the presence of meconium in both amniotic fluid and neonatal trachea and chest radiograms showing massive bilateral patchy infiltrates of the lung and respiratory failure [16]. Diagnosis of PPH was made by echocardiography [17]. TTN was defined as oxygen supplement requirement during the first 6 h of life that decreases during the subsequent 18 h, improvement in clinical condition within 6 h, and chest X-rays which were either normal or show reduced translucency, infiltrates, and hyperinflation of the lungs [18]. ARDS was diagnosed (after exclusion of infant respiratory distress syndrome, TTN, and congenital heart disease) as onset of respiratory failure in term infants from birth until 4 weeks of life with diffuse, bilateral, irregular opacities or infiltrates or complete opacification of the lungs which are not explained by local effusions or atelectasis or congenital lung anomalies [19]. A composite neonatal adverse outcome was defined as the occurrence of at least one of the following: need of resuscitation in the delivery room, blood cord pH < 7.100, occurrence of MAS, PPH, TTN, ARDS, HIE, and sepsis. 

For each pregnancy, we recorded maternal age, parity, smoking, positive recto-vaginal GBS swab, premature rupture of membranes (PROM) >18 h, clinical chorioamnionitis, hypertensive disorders of pregnancy, maternal diabetes, polyhydramnios, oligohydramnios, placental abruption, cord prolapse, and uterus rupture. Moreover, we reported labor induction and duration; occurrence of fever (>38 °C); normal, indeterminate, and abnormal cardiotocography (CTG) [20]; mode of delivery; occurrence of trial of labor after a previous cesarean delivery (TOLAC); and epidural analgesia. Clinical chorioamnionitis was defined as the presence of fever with one or more of the following: maternal leukocytosis >15,000/mm^3^, uterine tenderness, fetal tachycardia, or foul-smelling amniotic fluid).

### 2.4. Statistical Analysis

Clinical characteristics of infants were described as mean and standard deviation, median and range, or rate and percentage. Normality of data distribution was assessed by Shapiro–Wilk test. Parametric continuous variables were analyzed by the Student’s “t” test or by Wilcoxon rank-sum test in case of deviation from normality assumptions. Categorical variables were compared using the Χ^2^ test. *p* < 0.05 was considered statistically significant.

The primary endpoint of the study was to assess the possible correlation between a composite adverse neonatal outcome with MSAF of different thickness, while the secondary endpoint was to evaluate the possible correlation between delivery with grade 2 and 3 MSAF and the occurrence of some maternal, antepartum, and labor characteristics and mode of delivery. Therefore, a multivariable logistic regression analysis was performed to evaluate the possible independent effect of most important variables, such as grade 1, 2, and 3 SMAF, maternal age, hypertensive disorders of pregnancies, maternal diabetes, PROM >18 h, clinical chorioamnionitis, oligohydramnios, indeterminate plus abnormal CTG, labor induction, and operative delivery on the risk of composite adverse neonatal outcome. Moreover, a second multivariable logistic regression analysis was performed to evaluate the effects of maternal parity and diabetes, labor induction and duration, indeterminate CTG, cesarean section, and epidural analgesia on the risk of delivery with grade 2 and 3 MSAF. Effect estimates were expressed as odds ratio (OR) with profile likelihood-based 95% confidence limits.

Post hoc analyses demonstrated that the study has 100% power to detect a 25% difference in rates of composite adverse outcome in infants delivered with clear amniotic fluid vs. infants delivered with stage 3 MSAF (1 vs. 26%).

## 3. Results

We studied a total of 400 infants. Maternal characteristics and antepartum and intrapartum events are reported in Table 1. Parity was higher and both twinning and diabetes were more frequent in MSAF deliveries than in controls, with these differences being more evident in the grade 3 MSAF group in comparison with clear amniotic fluid deliveries. (Table 1 and Table 2) 

Labor induction was more frequent in grade 2 and 3 MSAF deliveries than in controls, while labor duration was longer in MSAF grades than in clear amniotic fluid deliveries. Grade 3 MSAF deliveries were associated with a higher frequency of indeterminate CTG, cesarean section, and operative delivery than controls, while epidural analgesia was more frequent in MSAF grades than in clear amniotic fluid deliveries. (Table 3 and Table 4) 

Infants with grade 2 and 3 MSAF had higher gestational age and required resuscitation in the delivery room more frequently than controls. Consistently, the occurrence of a cord blood pH < 7.100 was greater in these patients, who also showed a greater need of admission in NICU and greater occurrence of TTN. It is interesting to note that two patients developed MAS (both in the stage 3 MSAF group), while none developed HIE. (Table 5 and Table 6)

With regard to the primary endpoint of the study, multivariable stepwise logistic regression analysis showed that the risk of composite adverse neonatal outcome (the occurrence of at least one of the following: need of resuscitation in the delivery room, umbilical cord pH ≤ 7.100, occurrence of MAS, PPH, TTN, ARDS, HIE, and sepsis) was higher in infants with grade 2 (OR 16.82, 95% Cl 2.12–33.52; *p* = 0.008) and grade 3 (OR 33.79, 95% Cl 4.24–69.33; *p* <0.001) MSAF, clinical chorioamnionitis (OR 69.60, 95% Cl 5.15–94.68; *p* = 0.001), oligohydramnios (OR 4.47, 95% Cl 1.14–17.61; *p* = 0.032), and lower after-labor induction (OR 0.39, 95% Cl 0.17–0.86; *p* = 0.020). Conversely, grade 1 SMAF, maternal age, hypertensive disorders of pregnancies, maternal diabetes, PROM >18 h, CTG, and operative delivery did not affect the risk of composite adverse neonatal outcome (Table 7).

With regard to the secondary endpoint of the study, multivariable stepwise logistic regression analysis showed that the risk of delivery with grade 2 and 3 MSAF was increased by labor duration (OR 1.01, 95% Cl 1.00–1.01; *p* = 0.002), occurrence of indeterminate CTG (OR 2.72, 95% Cl 1.52–4.85; *p* <0.001), and epidural analgesia (OR 1.76, 95% Cl 1.07–2.89; *p* = 0.027). Conversely, parity, maternal diabetes, labor induction, operative delivery, and caesarean section did not affect the risk of delivery with grade 2 and 3 MSAF. (Table 8) 

## 4. Discussion

In this study, we assessed the hypothesis that there was a correlation between birth in the presence of MSAF and the outcomes of term infants born after a regular pregnancy and that this correlation depended on the grade of thickness of MSAF. We found that delivery with grade 2 and 3 MSAF, clinical chorioamnionitis, and oligohydramnios increased the risk of composite adverse neonatal outcome, while labor induction decreased it. 

Our results confirm the findings of Gluck et al., who recently documented in a large retrospective study the correlation between an adverse neonatal outcome and different thickness levels of MSAF [13]. In this study, the staging of MSAF (light, intermediate, and heavy) was very similar to ours, while some differences distinguished the neonatal composite outcomes, which also included the need for blood transfusion, need for phototherapy, necrotizing enterocolitis, intraventricular hemorrhage, hypoxic-ischemic encephalopathy, periventricular leukomalacia, seizures, hypoglycemia, hypothermia, and death. However, in agreement with our study, Gluck et al. found that intermediate (OR 1.51, 95% Cl 1.18–1.93; *p* = 0.001) and heavy (OR 2.42, 95% Cl 1.56–3.75; *p* < 0.001) MSAF significantly predict an adverse outcome, while light meconium did not [13]. In disagreement with our study and previous investigations [10], the occurrence of HIE in the work of Gluck et al. was higher in the intermediate and heavy MSAF groups than in the clear amniotic fluid group, but most likely it depends by the population size which was much larger [13]. Conversely, the occurrence of MAS was found to be correlated with the thickness of MSAF both in our study and in other previous works [10,13]. The mechanism by which grade 2 and 3 MSAF increase the risk of adverse neonatal outcomes is represented by their common etiologic factors, since fetal hypoxia can induce both an increase in intestinal peristalsis and relaxation of the anal sphincters, leading to intrauterine passage of meconium in the amniotic fluid and the need for resuscitation and metabolic acidosis in the delivery room and the occurrence of neonatal complications such as MAS, PPH, TTN, ARDS, and HIE. Therefore, it can be speculated that the grading of MSAF is a biomarker of fetal hypoxia and it increases proportionally to the severity of hypoxia as suggested by the increase of risk of adverse neonatal outcome in infants with grade 3 MSAF in comparison with infants with grade 2 MSAF. On the other hand, the lack of risk in presence of grade 1 MSAF seems to suggest that in these infants MSAF may represent, at least in many cases, the physiologic effect of fetal gastrointestinal maturation and motility or the fetal response to mild hypoxic stress.

In our study, we also found that clinical chorioamnionitis and oligohydramnios increase the risk of adverse neonatal outcome independently from MSAF. These results confirm previous studies which report the role of chorioamnironitis in promoting inflammatory fetal response and an increased risk of pneumonia, sepsis, cerebral palsy, and perinatal death in term infants [21,22,23]. Conversely, the role of oligohydramnios is more debatable since some studies did not show any effect on perinatal outcome [24], while others demonstrated it, such as an increased rate of low birth infants [25]. 

Among risk factors for delivery with grade 2 and 3 MSAF, we confirmed a positive correlation with the duration of labor which is expected considering that the physiopathological mechanisms of MSAF can be favored by its prolongation [26]. Similarly, the correlation between abnormal CTG and development of grade 2 and 3 MSAF confirms the results of previous studies [27,28] and can be explained by the fact that both are expressions of fetal distress. In agreement with Kim et al. [29] and Salameh et al. [30], we confirmed that epidural analgesia increased the risk of grade 2 and 3 MSAF, but this correlation requires further confirmation because other authors have found conflicting results [31,32]. 

Limitations of our study include its retrospective design and the fact that the severity of meconium staining was graded by visual assessment which may have possible sub-optimal accuracy with intra- and inter-observer variability [6,13]. However, the single-center design of the study and its limited duration can support the homogeneity of MSAF grading. On the other hand, it has been reported that underestimation of the severity of meconium staining is more likely than overestimation [6,13], and, therefore, any inaccuracies should be of little relevance to the study results. We could not compare our findings with those of recent studies on neonatal outcome of infants born with MSAF because they classified meconium consistency in only two categories: thick (amniotic fluid dark green in color and with a pea-soup consistency) or thin (amniotic fluid lightly stained a yellow or greenish color) [14,15].

## 5. Conclusions

We found that there is a positive correlation between the severity of meconium staining and thickness and the outcomes of term infants born after a non-eventful pregnancy. In fact, our results show that delivery with grade 2 and 3 MSAF is independently correlated with an increased risk of adverse neonatal outcome. These findings support the usefulness of MSAF evaluation and grading during labor in order to plan for the presence of a neonatologist at delivery for immediate and proper neonatal care. 

## Figures and Tables

**Table 1:** Maternal characteristics in deliveries with clear amniotic fluid (Controls) or with grade 1, 2, and 3 meconium-stained amniotic fluid. Mean ± SD, or median and (range), or rate and (%).

	Maternal Age (Y)	Parity	Twin	Smoking	Positive GBS Swab
Controls	33 ± 5	0 (0–5)	4 (4)	2 (2)	34 (34)
Grade 1	34 ± 5	0 (0–4)	3 (3)	5 (5)	23 (23)
Grade 2	35 ± 5	0 (0–2) *	1 (1)	2 (2)	31 (31)
Grade 3	35 ± 6	0 (0–5) **	0 ***	2 (2)	35 (35)

* *p* = 0.015 vs. controls; ** *p* = 0.017 vs. Grade 2; *** *p* = 0.043 vs. controls.

**Table 2:** Maternal characteristics in deliveries with clear amniotic fluid (Controls) or with grade 1, 2. and 3 meconium-stained amniotic fluid. Mean ± SD, or median and (range), or rate and (%).

	PROM > 18 h	C. Chorio.	HDP	Maternal Diabetes	Poly- Hydramnios	Oligo- Hydramnios	Placental Abruption	Cord Prolapse	Uterus Rupture
Controls	8 (8)	2 (2)	2 (2)	1 (1)	4 (4)	6 (6)	0	0	1 (1)
Grade 1	5 (5) *	1 (1)	8 (8)	13 (13) **	5 (5)	4 (4)	0	0	0
Grade 2	17 (17)	2 (2)	4 (4)	6 (6)	3 (3)	1 (1)	1 (1)	1 (1)	0
Grade 3	8 (8)	0	8 (8)	15 (15) **°	4 (4)	2 (2)	0	0	0

PROM: Premature rupture of membrane; C. Chorio.: clinical chorioamnionitis; HDP: hypertensive disorders of pregnancy. * *p* = 0.007 vs. grade 2; ** *p* <0.001 vs. controls; ° *p* = 0.038 vs. grade 2.

**Table 3:** Labor characteristics in case of clear amniotic fluid (Controls) or with grade 1, 2, and 3 meconium-stained amniotic fluid. Mean ± SD or rate and (%).

	Labor induction		Labor Duration			Fever	CTG		
	DVI	Oxytocin	Total	Stage 1	Stage 2		Normal	Indeterminate	Abnormal
Controls	6 (6)	16 (16)	158 ± 108	103 ± 74	50 ± 43	7 (7)	92 (92)	8 (8)	0
Grade 1 *	10 (10)	18 (18)	205 ± 129 ^c^	144 ± 93 ^c^	61 ± 45	13 (13)	84 (84)	14 (14)	0
Grade 2 **	16 (16) ^a^	20 (20)	205 ± 133 ^d^	133 ± 97 ^f^	66 ± 51	16 (16) ^gh^	79 (79)	17 (17)	0
Grade 3 *	12 (12)	28 (28) ^b^	230 ± 111 ^e^	152 ± 90 ^c^	68 ± 48	7 (7)	47 (47) ^cil^	41 (41) ^il^	0

DVI: Dipronostone vaginal insert; CTG: cardiotocography. ^a^ *p* = 0.024 vs. controls; ^b^ *p* = 0.041 vs. controls; ^c^ *p* < 0.001 vs. controls; ^d^ *p* = 0.007 vs. controls.; ^e^ *p* < 0.001; ^f^ *p* = 0.015; ^g^ *p* = 0.046 vs. controls; ^h^ *p* = 0.046 vs. grade 2; ^i^ *p* < 0.001 vs. grade 1; ^l^ *p* < 0.001 vs. grade 2. * CTG was evaluated in 98 deliveries; ** CTG was evaluated in 96 deliveries.

**Table 4:** Mode of delivery in case of clear amniotic fluid (Controls) or with grade 1, 2, and 3 meconium-stained amniotic fluid. Mean ± SD or rate and (%).

	Cesarean Section	Operative Delivery	TOLAC	Epidural Analgesia
Controls	18 (18)	7 (7)	2 (2)	20 (20)
Grade 1	20 (20)	6 (6)	4 (4)	47 (47) ^e^
Grade 2	15 (15)	12 (12)	1 (1)	46 (46) ^e^
Grade 3	32 (32) ^ab^	19 (19) ^cd^	6 (6)	58 (58) ^e^

TOLAC: trial of labor after a previous cesarean delivery. ^a^ *p* = 0.022 vs. controls; ^b^ *p* = 0.005 vs. grade 2; ^c^ *p* = 0.012 vs. control; ^d^ *p* = 0.005 vs. grade 2; ^e^ *p* < 0.001 vs. controls.

**Table 5:** Clinical characteristics of infants delivered with clear amniotic fluid (Controls) or with grade 1, 2, and 3 meconium-stained amniotic fluid. Mean ± SD, or median and (range), or rate and (%).

	Controls	Grade 1	Grade 2	Grade 3
Gestational age (wks)	39.5 ± 1.3	39.8 ± 1.2	40.1 ± 1.1 ^a^	40.2 ± 1.0 ^ab^
Birth weight (g)	3316 ± 449	3296 ± 409	3318 ± 399	3412 ± 383 ^c^
Intrauterine growth restriction	1 (1)	2 (2)	3 (3)	2 (2)
Male	50 (50)	44 (44)	49 (49)	54 (54)
Apgar’s score				
at 1 min	9 (7–10)	9 (7–10)	9 (7–10)	9 (0–10)
at 5 min	10 (8–10)	10 (8–10)	10 (8–10)	9 (0–10)
Need of resuscitation in the DR	1 (1)	6 (6)	7 (7) ^d^	20 (20) ^aef^
CPAP/PPV	1 (1)	6 (6)	7 (7) ^d^	20 (20) ^aef^
Mechanical ventilation	0	0	0	2 (2)
Adrenaline	0	0	0	1 (1)
Blood cord pH	7.26 ± 0.81	7.22 ± 0.09	7.26 ± 0.89	7.22 ± 0.88
Blood cord pH < 7.100	0	0	5 (5) ^g^	8 (8) ^hi^
Blood cord SBE	−1.1	−1.4	−1.7	−1.7
Blood cord lactate (mmml/L)	4.3 ± 1.8	5.0 ± 2.0	5.0 ± 2.3	12.5 ± 6.5 ^alm^

DR: delivery room; CPAP: continuous positive airway pressure; PPV: positive pressure ventilation; SBE: standard base excess. ^a^ *p* < 0.001 vs. controls; ^b^ *p* = 0.011 vs. grade 1; ^c^ *p* = 0.040 vs. grade 1; ^d^ *p* = 0.030 vs. grade 2; ^e^ *p* = 0.004 vs. grade 1; ^f^ *p* = 0.007 vs. grade 2; ^g^ *p* = 0.024 vs. controls; ^h^ *p* = 0.004 vs. control; ^i^ *p* = 0.024 vs. grade 1; ^l^ *p* < 0.001 vs. grade 1; ^m^ *p* < 0.001 vs. grade 2.

**Table 6:** Outcomes of infants delivered with clear amniotic fluid (Controls) or with grade 1, 2, and 3 meconium-stained amniotic fluid. Rate and (%) or mean ± SD.

	Controls	Grade 1	Grade 2	Grade 3
Admission in neonatal units	14 (14)	9 (9)	14 (14) ^a^	25 (25) ^abc^
Special care unit	14 (14)	5 (5)	9 (9)	10 (10)
Intensive care unit	0	4 (4) ^d^	5 (5) ^e^	15 (15) ^fg^
Meconium aspiration syndrome	0	0	0	2 (2)
Persistent pulmonary hypertension	0	0	0	3 (3)
Inhaled nitric oxide	0	0	0	3(34)
Transient tachypnea of the newborn	0 (0)	4 (4) ^h^	5 (5) ^i^	15 (15) ^ag^
Acute respiratory distress syndrome	0	2 (2)	1 (1)	6 (6) ^l^
Air leak	0	0	0	1 (1)
Hypoxic ischemic encephalopathy	0	0	0	3 (3)
Mild therapeutic hypothermia	0	0	0	3 (3)
Seizures	0	0	0	2 (2)
Sepsis	0	0	0	1 (1)
Jaundice requiring phototherapy	8 (8)	4 (4)	0 ^mn^	3 (3)
Stay in hospital duration (d)	3.5 ± 1.5	3.3 ± 1.6	3.4 ± 2.0	4.4 ± 3.5
Composite adverse outcome	1 (1)	6 (6)	14 (14) ^a^	26 (26) ^aco^

^a^ *p* < 0.001 vs. controls; ^b^ *p* = 0.049 vs. controls; ^c^ *p* < 0.001 vs. grade 2; ^d^ *p* = 0.043 vs. controls; ^e^ *p* = 0.024 vs. controls; ^f^ *p* < 0.001 vs. controls; ^g^ *p* = 0.018 vs. grade 2; ^h^ *p* = 0.034 vs. controls; ^i^ *p* = 0.024 vs. controls; ^l^ *p* = 0.013 vs. controls; ^m^ *p* = 0.004 vs. controls; ^n^ *p* = 0.044 vs. grade 1; ^o^ *p* = 0.034.

**Table 7:** The results of a multivariate regression analysis model for composite adverse neonatal outcome (need of resuscitation in the delivery room, pH < 7.100, meconium aspiration syndrome, persistent pulmonary hypertension, transient tachypnea of the newborn, hypoxic-ischemic encephalopathy, acute respiratory distress syndrome, sepsis.).

	OR	95% Cl	P
Grade 1 MSAF	4.35	0.48–39.18	0.189
Grade 2 MSAF	16.82	2.12–33.52	0.008
Grade 3 MSAF	33.79	4.24–69.33	<0.001
Maternal age	0.99	0.93–1.06	0.189
Hypertensive disorders of pregnancies	1.27	0.29–5.57	0.750
Maternal diabetes	1.78	0.76–4.17	0.750
PROM	1.12	0.35–3.57	0.843
Clinical chorioamnionitis	69.60	5.15–940.68	0.001
Oligohydramnios	4.47	1.14–17.61	0.032
Indeterminate CTG	1.26	0.70–2.24	0.439
Labor induction	0.39	0.17–0.86	0.020
Operative delivery	0.56	0.18–1.75	0.317

MSAF: meconium-stained amniotic fluid; PROM: premature rupture of membrane; CTG: cardiotocography.

**Table 8:** The results of a multivariate regression analysis model for delivery with grade 2 and 3 meconium-stained amniotic fluid (MSAF).

	OR	95% Cl	P
Parity	0.90	0.66–1.22	0.491
Maternal diabetes	1.34	0.60–2.99	0.469
Labor induction	1.22	0.75–1.99	0.420
Labor duration	1.01	1.00–1.01	0.002
Indeterminate CTG	2.72	1.52–4.85	<0.001
Operative delivery	1.01	0.53–1.94	0.973
Cesarean section	0.910	0.52–1.59	0.734
Epidural analgesia	1.76	1.07–2.89	0.027

CTG: cardiotocography.

## Data Availability

All relevant data are within the manuscript.
